# Supplementary material for: Laser flash melting cryo-EM samples to overcome preferred orientation
Source: Nat Methods. 2025 Aug 28;22(9):1880–6. doi: 10.1038/s41592-025-02796-y (PMC12446059; doi:10.1038/s41592-025-02796-y)
Supplement: Supplementary file 1 — Supplementary Figures 1–15 and Supplementary Tables 1–4. [file 41592_2025_2796_MOESM1_ESM.pdf]

nature methods

Supplementary information https://doi.org/10.1038/s41592-025-02796-y

Laser flash melting cryo-EM samples to overcome preferred orientation

In the format provided by the authors and unedited

This PDF file includes:

1 | Cryo-EM data processing – T20S proteasome

2 | Cryo-EM data processing – 50S ribosomal subunit

3 | Cryo-EM data processing – HIV-1 Envelope ectodomain protein

4 | Cryo-EM data processing – Hemagglutinin

5 | Simulation of the temperature evolution of the sample

6 | Cryo-EM data collection, refinement and validation statistics

7 | References

1 | Cryo-EM data processing T20S proteasome

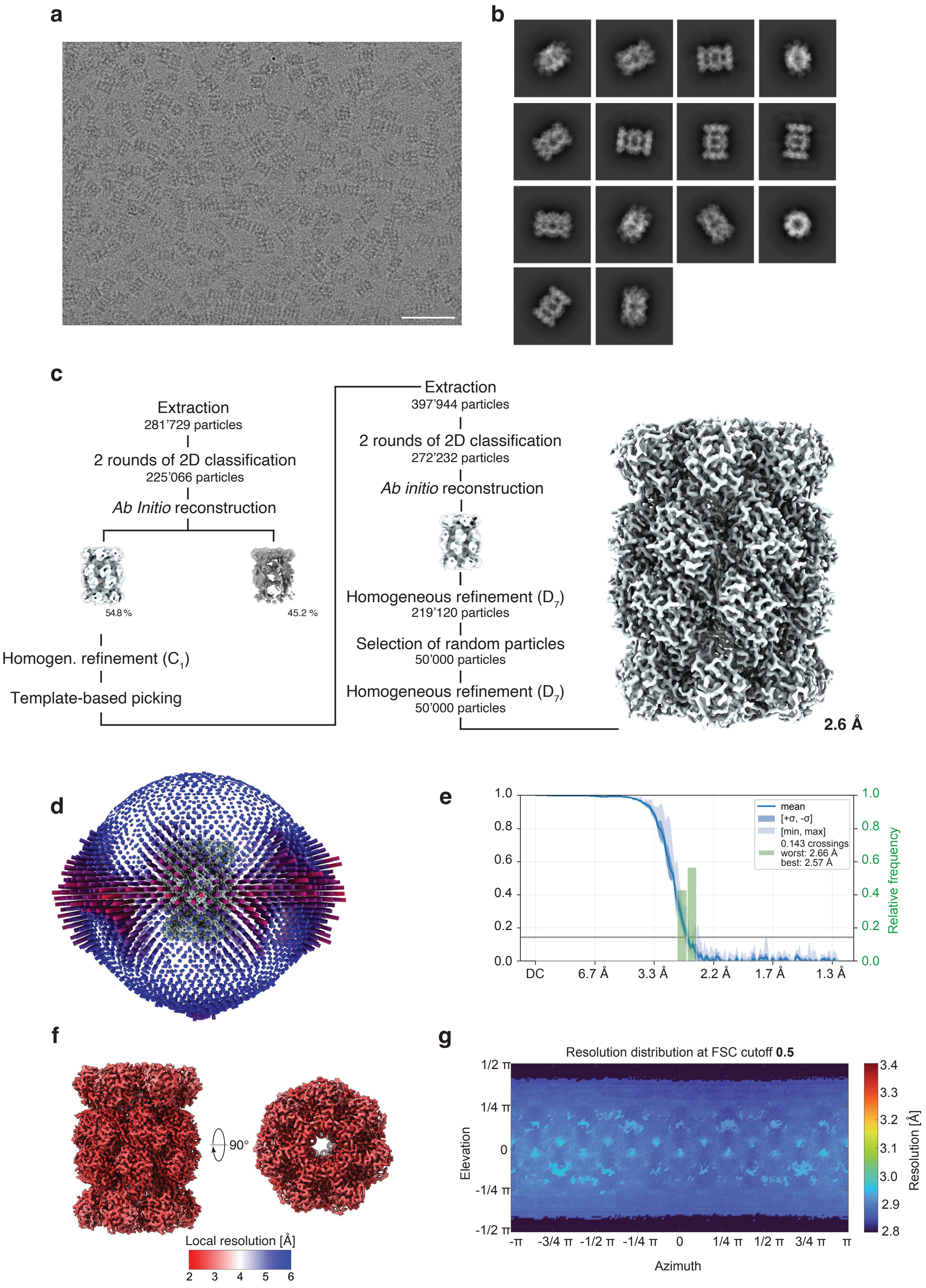

Supplementary Figure 1 | Cryo-EM data processing workflow for a conventional sample of the T20S proteasome. a Representative micrograph, revealing that side views of the proteasome are predominantly obtained. Scale bar, 500 Å. **b** 2D class averages, underlining the abundance of side views. **c** Data processing workflow in cryoSPARC. The symmetry applied in each step is indicated in parentheses. A resolution of 2.6 Å was obtained in the final reconstruction. The map is shown at a 5 σ. **d** Three-dimensional representation of angular distribution of the particles, with the color and height of the cylinders representing the frequency of observation (red: more abundant, blue: less abundant). **e** Plot of the 3DFSC with the 0.143 cutoff indicated (grey line). The mean FSC value is shown as a solid blue line, with dark blue shading corresponding to one standard deviation, and the light blue shading indicating the minimum and maximum values. The green bars represent a histogram of the resolution values obtained from the directional FSC. **f** Final map with the local resolution estimation indicated in color. **g** The resolution distribution plot shown at FSC cutoff of 0.5, as obtained from the 3DFSC job in cryoSPARC.

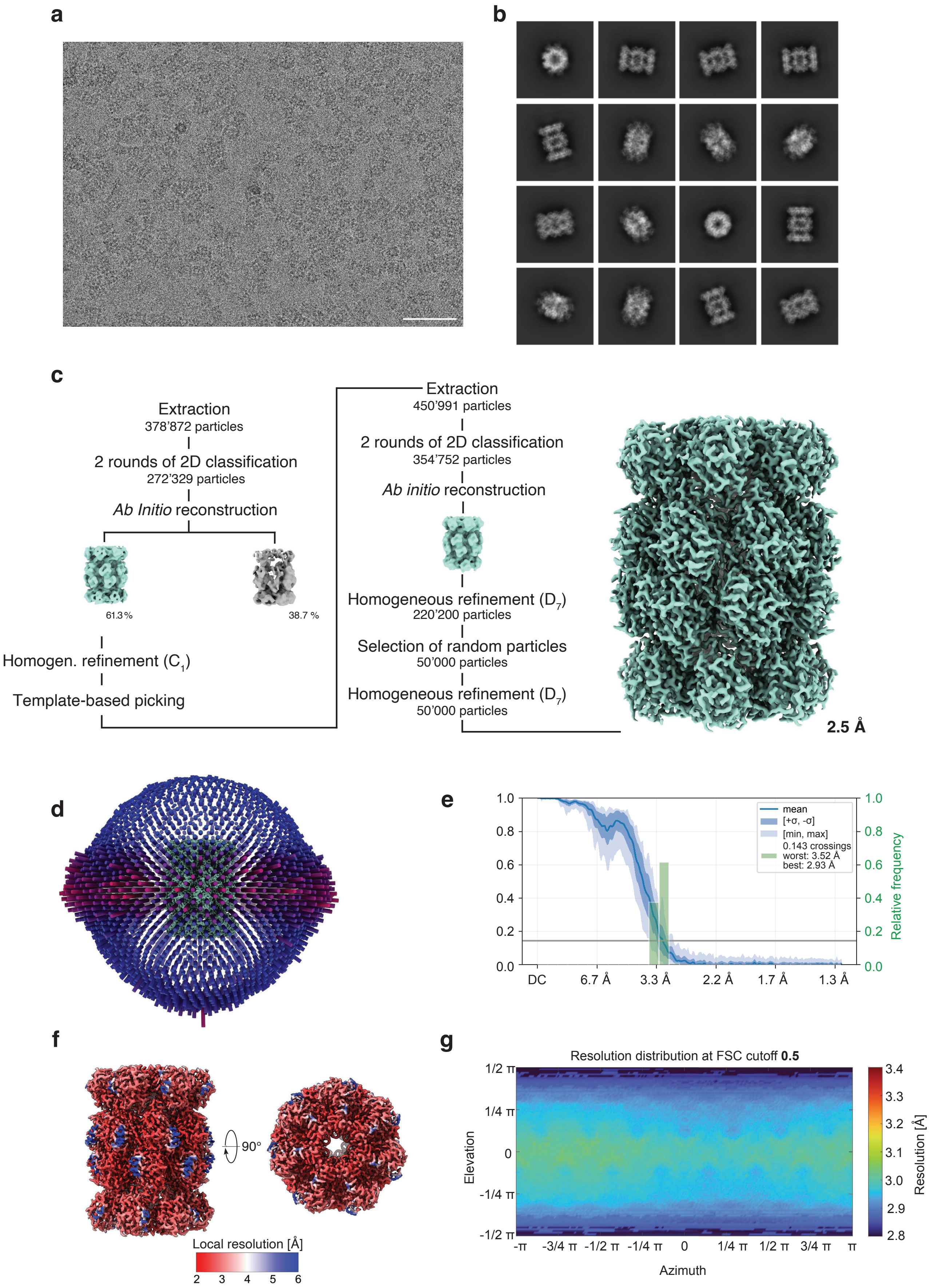
a
b
c
Extraction
378'872 particles
2 rounds of 2D classification
272'329 particles
Ab Initio reconstruction
61.3 %
38.7 %
Homogen. refinement (C1)
Template-based picking
Extraction
450'991 particles
2 rounds of 2D classification
354'752 particles
Ab initio reconstruction
Homogeneous refinement (D7)
220'200 particles
Selection of random particles
50'000 particles
Homogeneous refinement (D7)
50'000 particles
2.5 Å
d
e
mean
[+σ, -σ]
[min, max]
0.143 crossings
worst: 3.52 Å
best: 2.93 Å
Relative frequency
DC
6.7 Å
3.3 Å
2.2 Å
1.7 Å
1.3 Å
f
90°
Local resolution [Å]
2 3 4 5 6
g
Resolution distribution at FSC cutoff 0.5
Elevation
Azimuth
Resolution [Å]

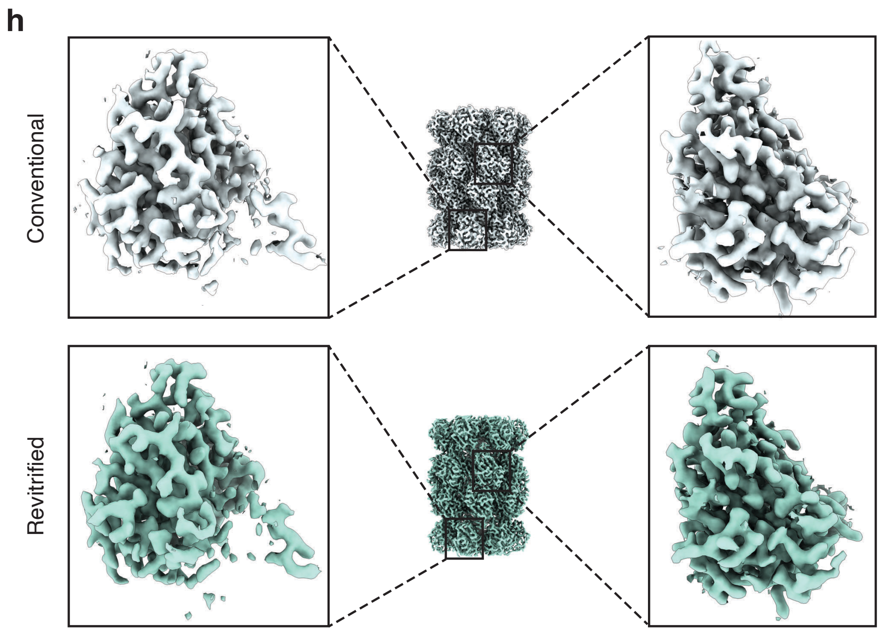

Supplementary Figure 2 | Cryo-EM data processing workflow for a revitrified sample of the T20S proteasome. a Representative micrograph. Scale bar 500 Å. **b** 2D class averages revealing more tilted views of the T20S proteasome. **c** Data processing workflow in cryoSPARC. The symmetry applied is indicated in parentheses. A resolution of 2.5 Å was obtained in the final reconstruction. The map is shown 5 σ. **d** Three-dimensional representation of angular distribution of the particles, with the color and height of the cylinders representing the frequency of observation (red: more abundant, blue: less abundant). **e** Plot of the 3DFSC with the 0.143 cutoff indicated (grey line). The mean FSC value is shown as a solid blue line, with dark blue shading corresponding to one standard deviation, and the light blue shading indicating the minimum and maximum values. The green bars represent a histogram of the resolution values obtained from the directional FSC. **f** Final map with the local resolution estimation indicated in color. **g** The resolution distribution plot shown at FSC cutoff of 0.5, as obtained from the 3DFSC job in cryoSPARC. The directional resolutions appear slightly worse for the revitrified, compared to the conventional sample. However, the differences are minimal (within 0.1 Å) and are therefore considered as noise. **h** Comparison of the reconstructions from the conventional sample with the revitrified sample.

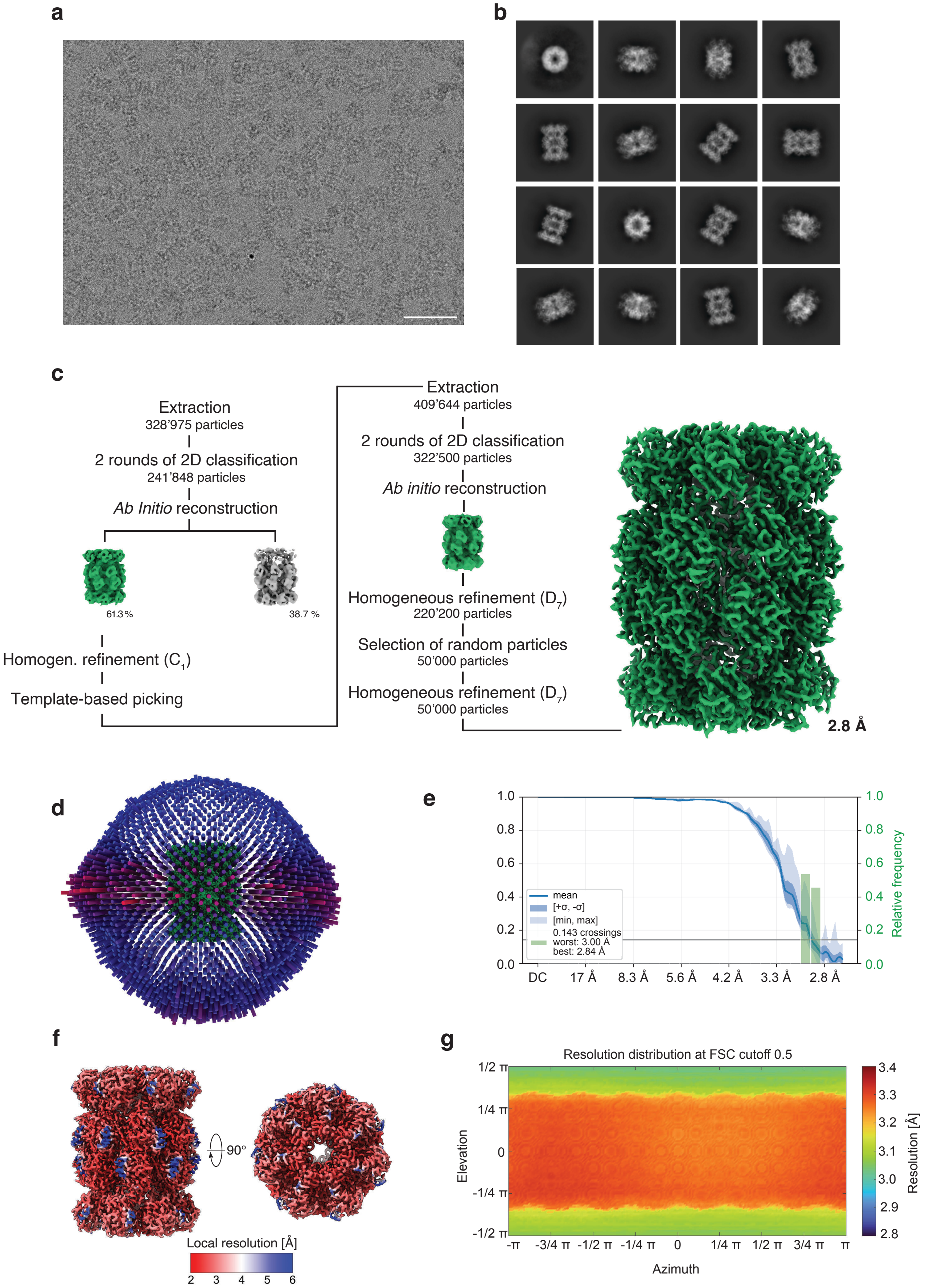
a
b
c
Extraction
328'975 particles
2 rounds of 2D classification
241'848 particles
Ab Initio reconstruction
61.3 %
38.7 %
Homogen. refinement (C1)
Template-based picking
Extraction
409'644 particles
2 rounds of 2D classification
322'500 particles
Ab initio reconstruction
Homogeneous refinement (D7)
220'200 particles
Selection of random particles
50'000 particles
Homogeneous refinement (D7)
50'000 particles
2.8 Å
d
e
mean
[+σ, -σ]
[min, max]
0.143 crossings
worst: 3.00 Å
best: 2.84 Å
Relative frequency
DC
17 Å
8.3 Å
5.6 Å
4.2 Å
3.3 Å
2.8 Å
f
90°
Local resolution [Å]
g
Resolution distribution at FSC cutoff 0.5
Elevation
Azimuth
Resolution [Å]

h

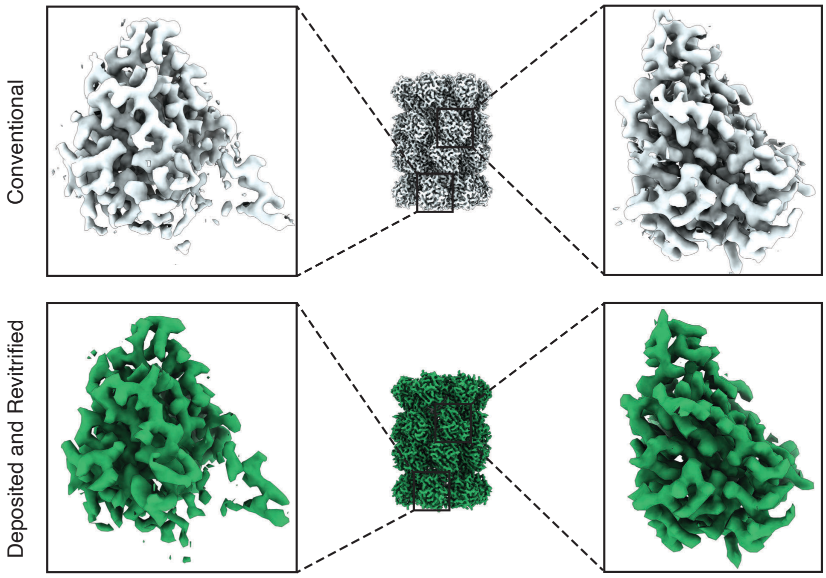

Supplementary Figure 3 | Cryo-EM data processing workflow for a deposited and revitrified sample of the T20S proteasome. a Representative micrograph. Scale bar 500 Å. **b** 2D class averages, revealing more tilted views. **c** Data processing workflow in cryoSPARC. The symmetry applied in each step is indicated in parentheses. A resolution of 2.8 Å was obtained in the final reconstruction. The map is contoured 5 σ. **d** Three-dimensional representation of angular distribution of the particles, with the color and the height of the cylinders representing the frequency of observation (red: more abundant, blue: less abundant). **d** Plot of the 3DFSC with the 0.143 cutoff indicated (grey line). The mean FSC value is shown as a solid blue line, with dark blue shading corresponding to one standard deviation, and the light blue shading indicating the minimum and maximum values. The green bars represent a histogram of the resolution values obtained from the directional FSC. **f** Final map with the local resolution estimation indicated in color. **g** The resolution distribution plot shown at FSC cutoff of 0.5, as obtained from the 3DFSC job in cryoSPARC. Due to the increase in ice thickness after deposition, the directional resolutions, as well as the overall resolution of the reconstruction, decreased minimally. **h** Comparison of the reconstructions from the conventional sample with the deposited and revitrified sample.

2 | Cryo-EM data processing – 50S ribosomal subunit

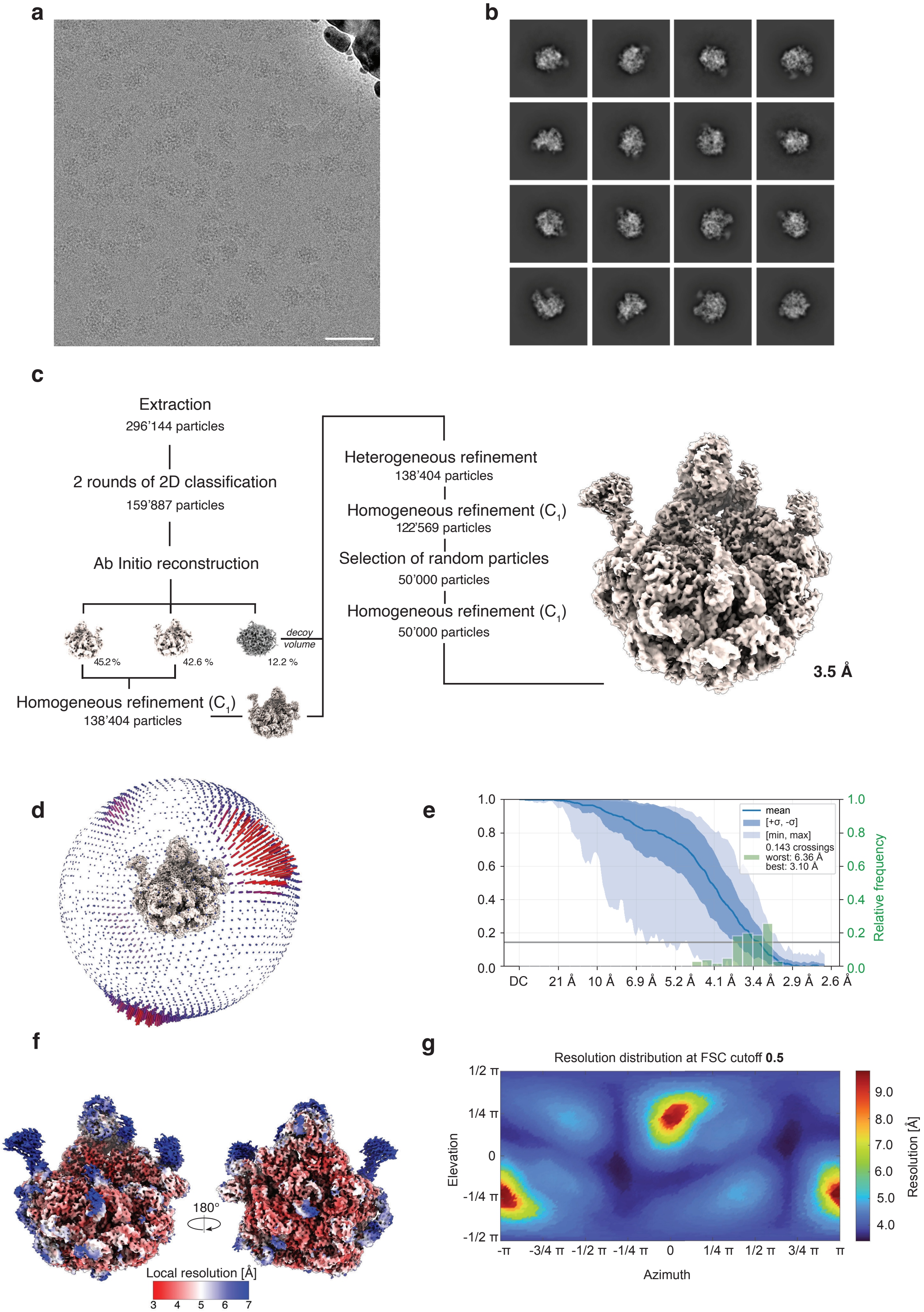

Supplementary Figure 4 | Cryo-EM data processing workflow for a conventional sample of the 50S ribosome. **a** Representative micrograph. Scale bar 100 Å. **b** 2D class averages **c** Data processing workflow in cryoSPARC. The symmetry applied in each step is indicated in parentheses. A resolution of 3.5 Å was obtained in the final reconstruction. The unsharpened map is shown at 3.5 σ. **d** Three-dimensional representation of angular distribution of the particles, with the color and the height of the cylinders representing the frequency of observation (red: more abundant, blue: less abundant). **d** Plot of the 3DFSC with the 0.143 cutoff indicated (grey line). The mean FSC value is shown as a solid blue line, with dark blue shading corresponding to one standard deviation, and the light blue shading indicating the minimum and maximum values. The green bars represent a histogram of the resolution values obtained from the directional FSC. **f** Final map with the local resolution estimation indicated in color. **g** The resolution distribution plot shown at FSC cutoff of 0.5, as obtained from the 3DFSC job in cryoSPARC. The plot shows regions with lower resolutions, corresponding to missing views in the angular distribution (panel d).

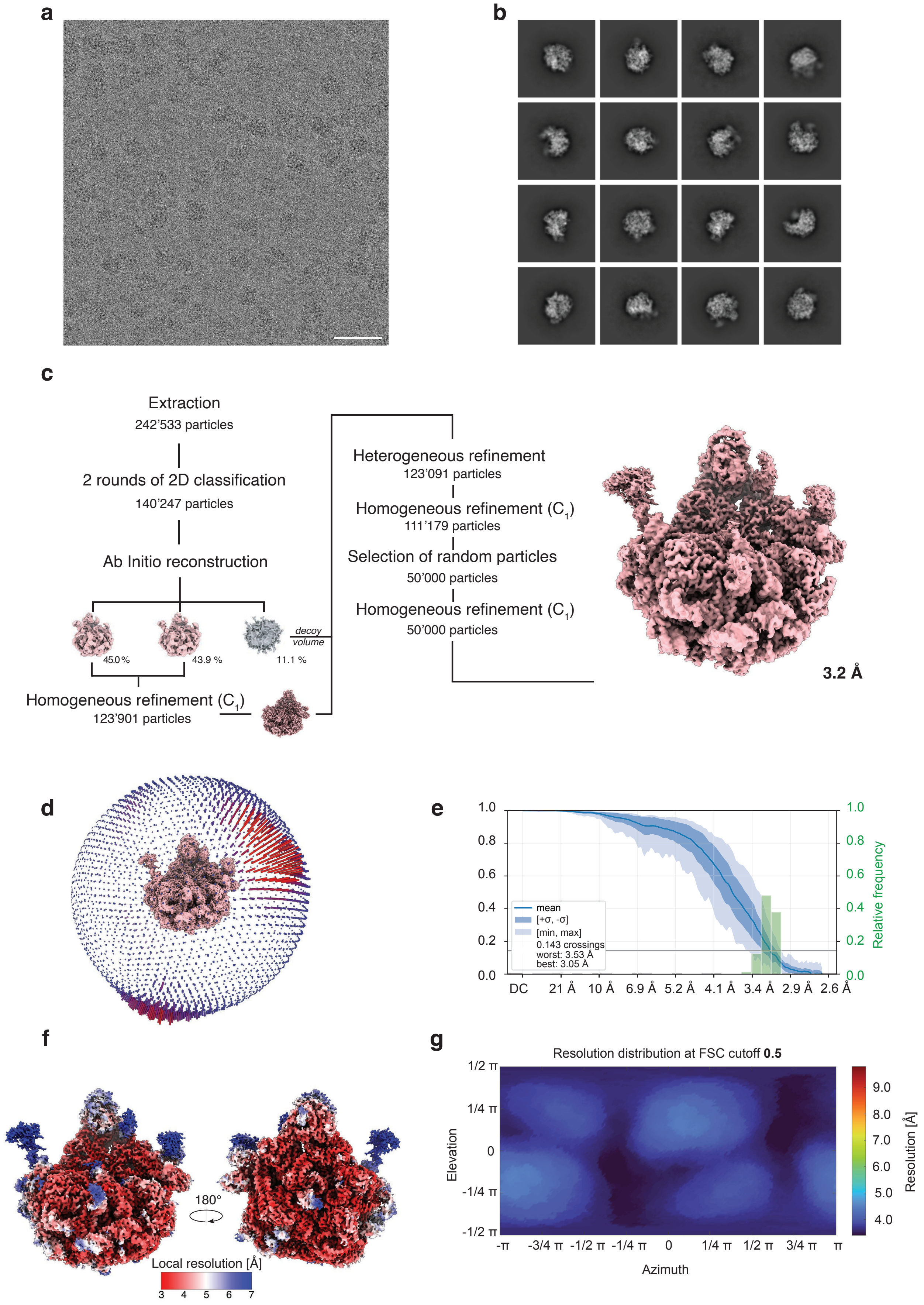
a
b
c
Extraction
242'533 particles
2 rounds of 2D classification
140'247 particles
Ab Initio reconstruction
45.0 %
43.9 %
11.1 %
decoy volume
Homogeneous refinement (C_1)
123'901 particles
Heterogeneous refinement
123'091 particles
Homogeneous refinement (C_1)
111'179 particles
Selection of random particles
50'000 particles
Homogeneous refinement (C_1)
50'000 particles
3.2 Å
d
e
mean
[+σ, -σ]
[min, max]
0.143 crossings
worst: 3.53 Å
best: 3.05 Å
DC 21 Å 10 Å 6.9 Å 5.2 Å 4.1 Å 3.4 Å 2.9 Å 2.6 Å
Relative frequency
f
180°
Local resolution [Å]
3 4 5 6 7
g
Resolution distribution at FSC cutoff **0.5**
Elevation
Azimuth
Resolution [Å]

h

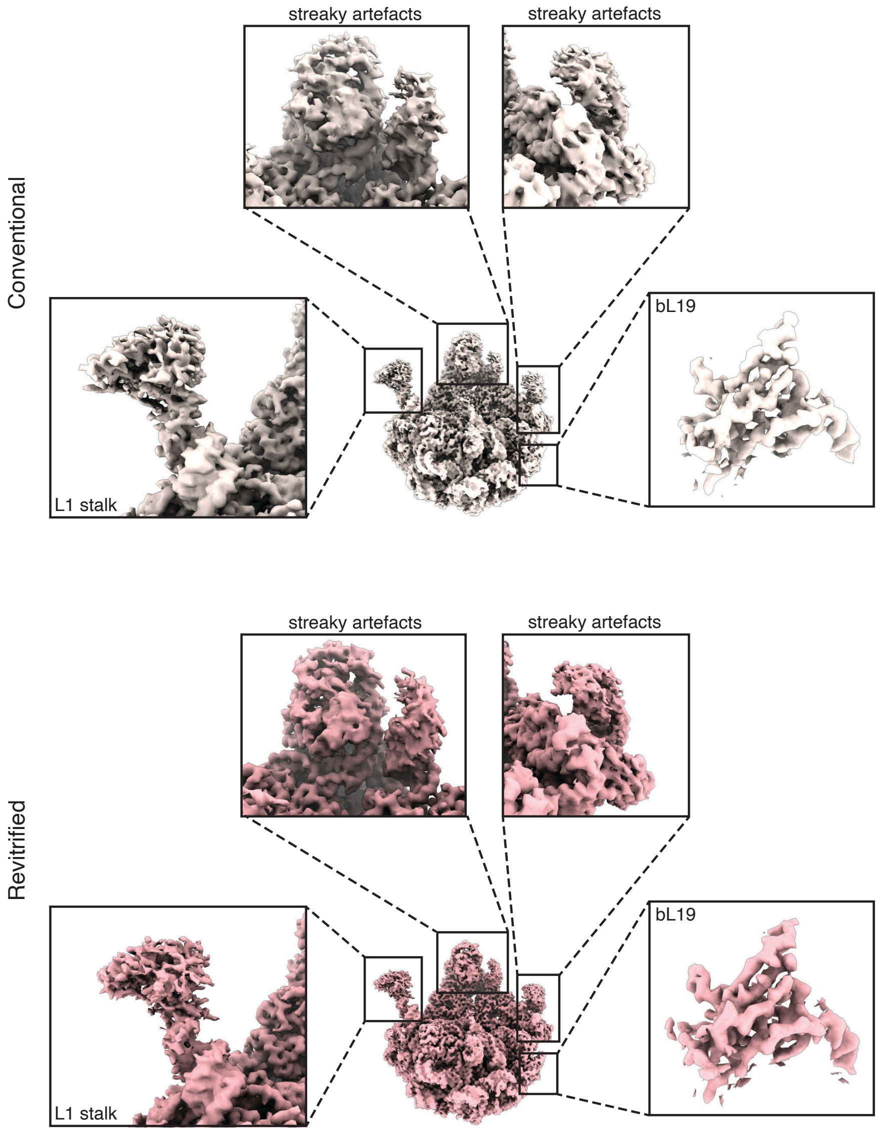

Supplementary Figure 5 | Cryo-EM data processing workflow for a revitrified sample of the 50S ribosome. a Representative micrograph. Scale bar 100 Å. **b** 2D class averages **c** Data processing workflow in cryoSPARC. The symmetry applied is indicated in parentheses. A resolution of 3.2 Å was obtained in the final reconstruction. The unsharpened map is shown at 3.5 σ. **d** Three-dimensional representation of angular distribution of the particles, with the color and height of the cylinders representing the frequency of observation (red: more abundant, blue: less abundant). **e** Plot of the 3DFSC with the 0.143 cutoff indicated (grey line). The mean FSC value is shown as a solid blue line, with dark blue shading corresponding to one standard deviation, and the light blue shading indicating the minimum and maximum values. The green bars represent a histogram of the resolution values obtained from the directional FSC. The more concise distribution of the minimum and maximum 0.143

crossing reflect the improvement of the preferred orientation of the 50S ribosomal subunit. **f** Final map with the local resolution estimation indicated in color. **g** The resolution distribution plot shown at FSC cutoff of 0.5, as obtained from the 3DFSC job in cryoSPARC. After revitrification, the resolution distribution also becomes more uniform. **h** Comparison of the reconstructions from the conventional sample with the revitrified sample. Top insets highlight the streaky artefacts, present in the conventional map due to preferred orientation. The same regions in the revitrified map reveal the disappearance of the streaky artefacts thanks to a better angular sampling obtained after revitrification. Bottom insets show the L1 stalk and the bL19 subunit (contoured at 6 σ) of the conventional and revitrified maps, respectively.

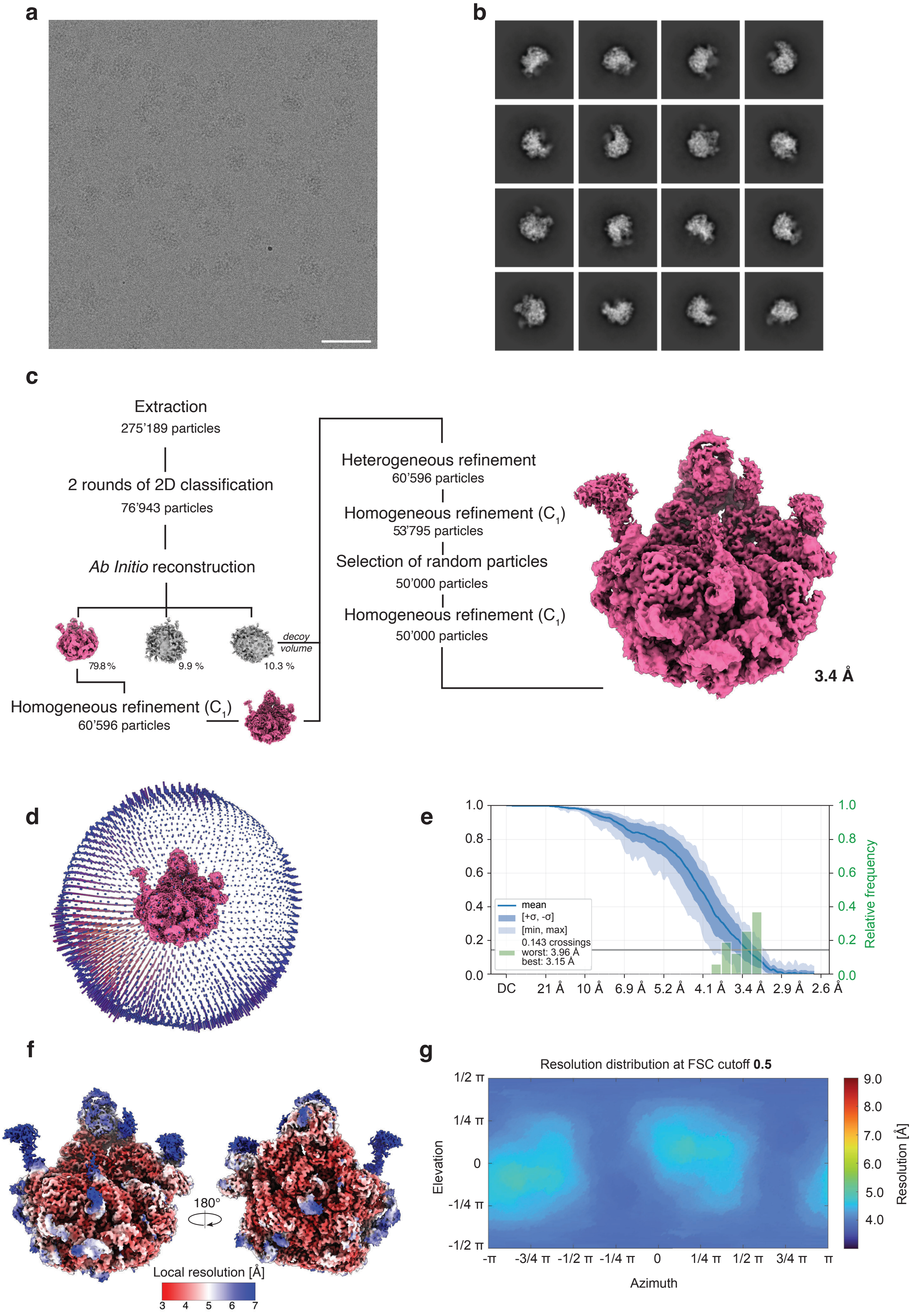
a
b
c
Extraction
275’189 particles
2 rounds of 2D classification
76’943 particles
Ab Initio reconstruction
79.8 %
9.9 %
10.3 %
decoy volume
Homogeneous refinement (C1)
60’596 particles
Heterogeneous refinement
60’596 particles
Homogeneous refinement (C1)
53’795 particles
Selection of random particles
50’000 particles
Homogeneous refinement (C1)
50’000 particles
3.4 Å
d
e
mean
[+σ, -σ]
[min, max]
0.143 crossings
worst: 3.96 Å
best: 3.15 Å
DC 21 Å 10 Å 6.9 Å 5.2 Å 4.1 Å 3.4 Å 2.9 Å 2.6 Å
Relative frequency
f
180°
Local resolution [Å]
3 4 5 6 7
g
Resolution distribution at FSC cutoff 0.5
Elevation
Azimuth
-π -3/4 π -1/2 π -1/4 π 0 1/4 π 1/2 π 3/4 π π
Resolution [Å]

h

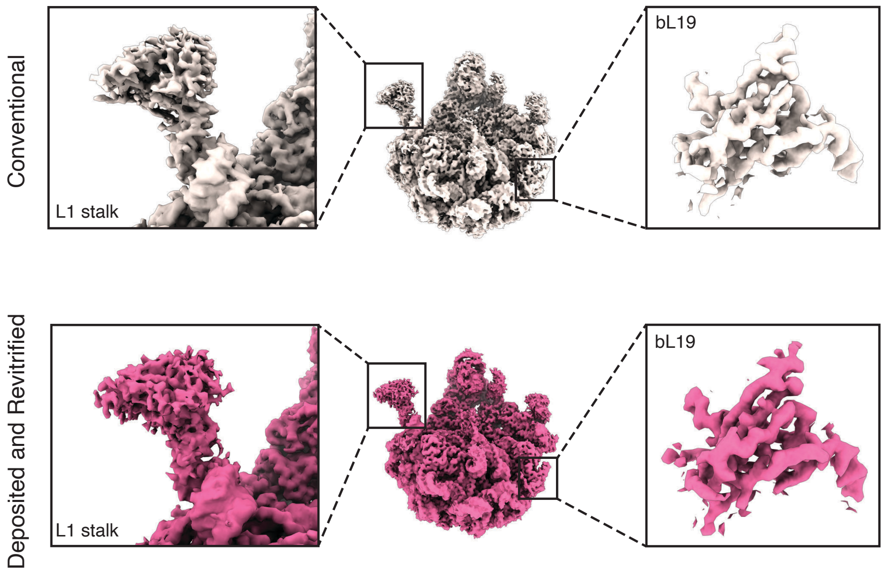

Supplementary Figure 6 | Cryo-EM data processing workflow for a deposited and revitrified sample of the 50S ribosome. a Representative micrograph. Scale bar 100 Å. **b** 2D class averages **c** Data processing workflow in cryoSPARC. The symmetry applied is indicated in parentheses. A resolution of 3.4 Å was obtained in the final reconstruction. The unsharpened map shown at 3.5 σ. **d** Three-dimensional representation of angular distribution of the particles, with the color and height of the cylinders representing the frequency of observation (red: more abundant, blue: less abundant). **e** Plot of the 3DFSC with the 0.143 cutoff indicated (grey line). The mean FSC value is shown as a solid blue line, with dark blue shading corresponding to one standard deviation, and the light blue shading indicating the minimum and maximum values. The green bars represent a histogram of the resolution values obtained from the directional FSC. The more concise distribution of the minimum and maximum 0.143 crossing reflect the improvement of the preferred orientation of the 50S ribosomal subunit. **f** Final map with the local resolution estimation indicated in color. **g** The resolution distribution plot shown at FSC cutoff of 0.5, as obtained from the 3DFSC job in cryoSPARC. Due to the increase in ice thickness after deposition, the directional resolutions, as well as the overall resolution of the reconstruction, decreased minimally compared to the reconstruction obtained from the revitrification-only experiment (Fig. S6g). Compared to the control, the directional resolution distribution improved (Fig. S5g). **h** Comparison of the reconstructions from the conventional sample with the deposited and revitrified sample. The bL19 subunit is contoured at 6 σ.

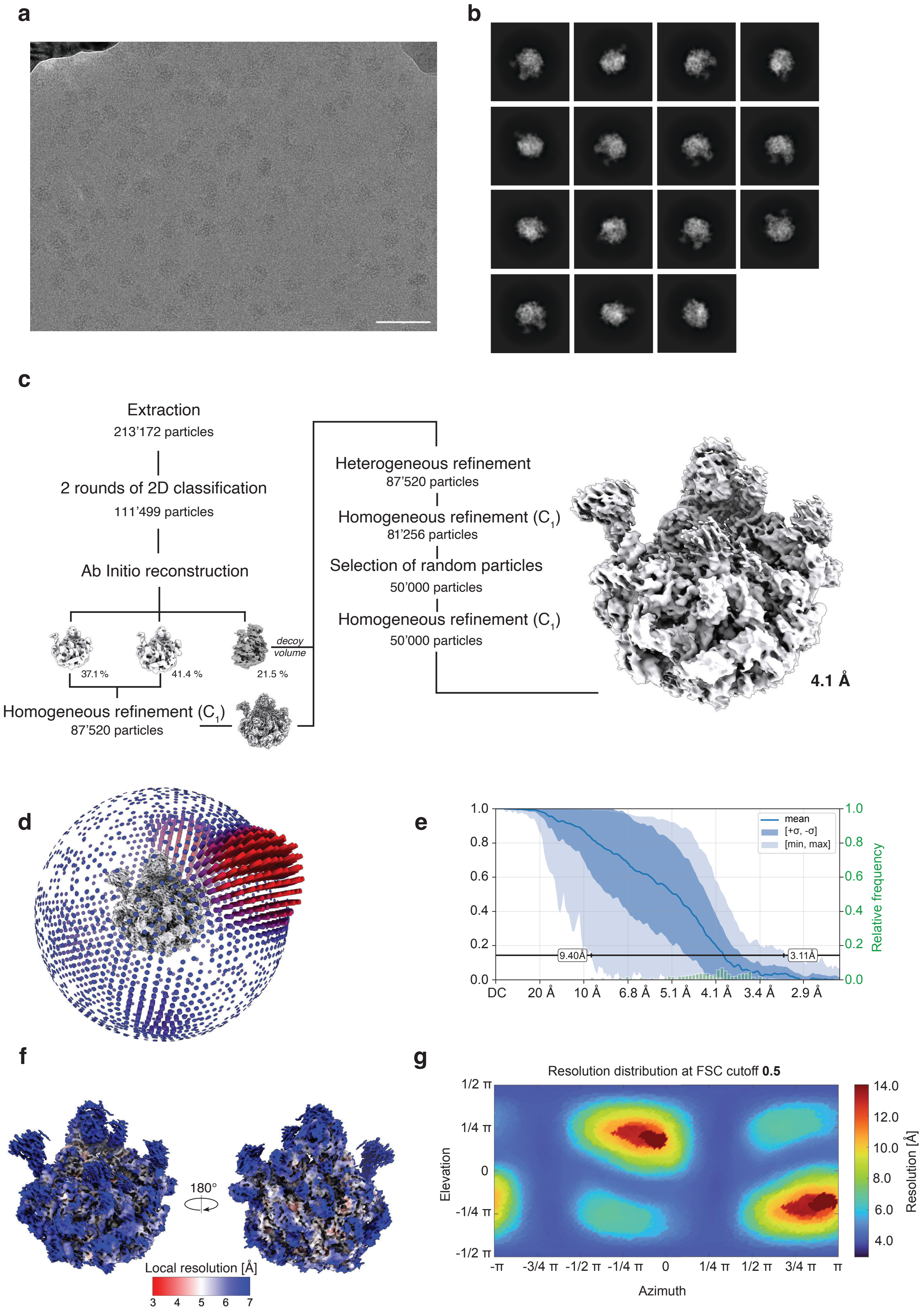
a
b
c
Extraction
213'172 particles
2 rounds of 2D classification
111'499 particles
Ab Initio reconstruction
37.1 %
41.4 %
21.5 %
decoy volume
Homogeneous refinement (C1)
87'520 particles
Heterogeneous refinement
87'520 particles
Homogeneous refinement (C1)
81'256 particles
Selection of random particles
50'000 particles
Homogeneous refinement (C1)
50'000 particles
4.1 Å
d
e
mean
[+σ, -σ]
[min, max]
Relative frequency
9.40Å
3.11Å
DC
20 Å
10 Å
6.8 Å
5.1 Å
4.1 Å
3.4 Å
2.9 Å
f
180°
Local resolution [Å]
3
4
5
6
7
g
Resolution distribution at FSC cutoff 0.5
Elevation
Azimuth
Resolution [Å]

Supplementary Figure 7 | Cryo-EM data processing workflow for a conventional sample of the 50S ribosome, used as the control for shaped pulse revitrification. a Representative micrograph. Scale bar 500 Å. **b** 2D class averages **c** Data processing workflow in cryoSPARC. The symmetry applied is indicated in parentheses. A resolution of 4.1 Å was obtained in the final reconstruction. The unsharpened map is shown at 3.5 σ. **d** Three-dimensional representation of angular distribution of the particles, with the color and height of the cylinders representing the frequency of observation (red: more abundant, blue: less abundant). **e** Plot of the 3DFSC with the 0.143 cutoff indicated (grey line). The mean FSC value is shown as a solid blue line, with dark blue shading corresponding to one standard deviation, and the light blue shading indicating the minimum and maximum values. The green bars represent a histogram of the resolution values obtained from the directional FSC. The broad distribution of the minimum and maximum 0.143 crossing underline the preferred orientation of the 50S ribosomal subunit. **f** Final map with the local resolution estimation indicated in color. **g** The resolution distribution plot shown at FSC cutoff of 0.5, as obtained from the 3DFSC job in cryoSPARC. The plot shows regions with lower resolutions, corresponding to missing views in the angular distribution (panel d).

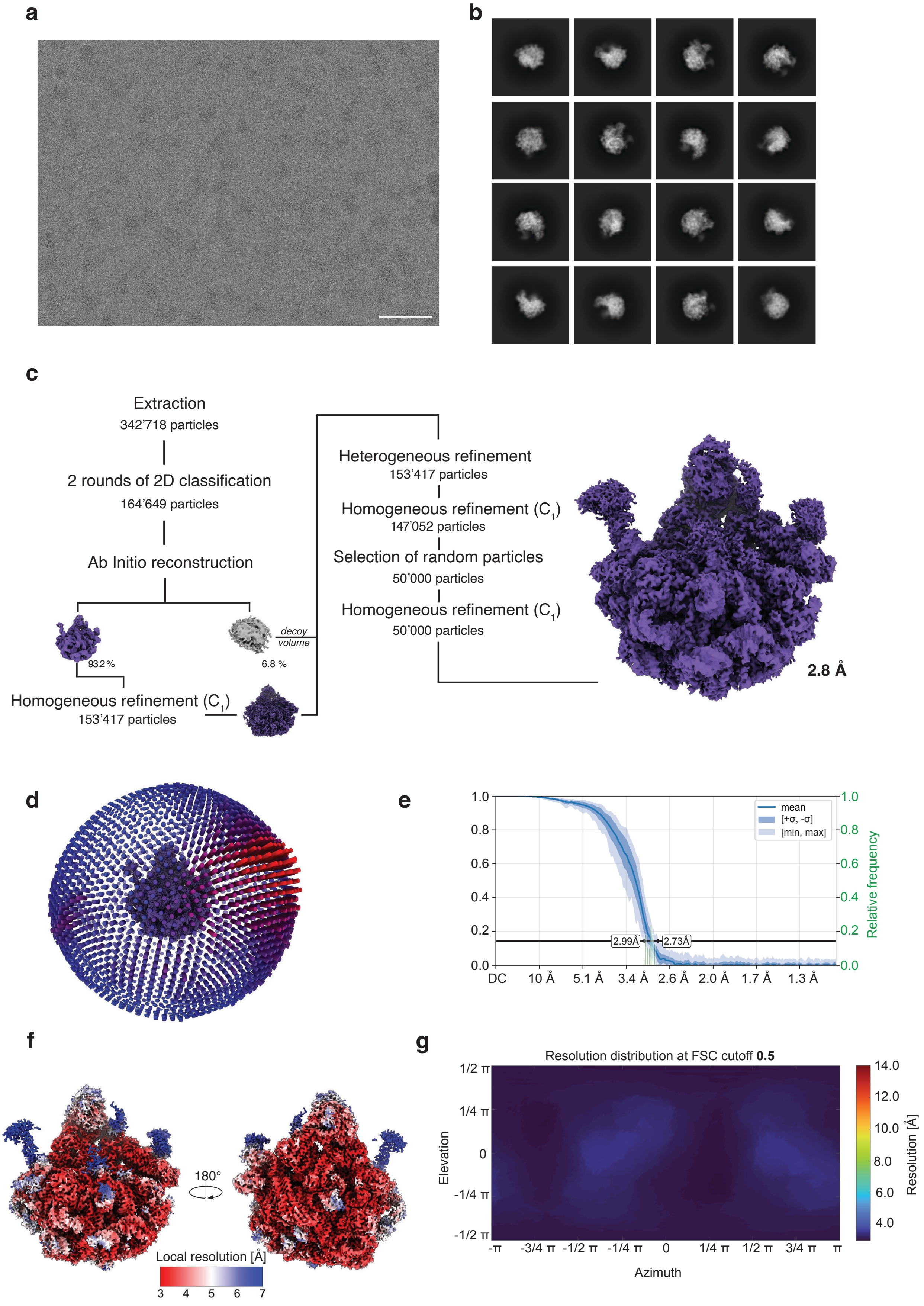
a
b
c
Extraction
342’718 particles
2 rounds of 2D classification
164’649 particles
Ab Initio reconstruction
93.2 %
decoy volume
6.8 %
Homogeneous refinement (C1)
153’417 particles
Heterogeneous refinement
153’417 particles
Homogeneous refinement (C1)
147’052 particles
Selection of random particles
50’000 particles
Homogeneous refinement (C1)
50’000 particles
2.8 Å
d
e
mean
[+σ, -σ]
[min, max]
Relative frequency
2.99Å
2.73Å
DC 10 Å 5.1 Å 3.4 Å 2.6 Å 2.0 Å 1.7 Å 1.3 Å
f
180°
Local resolution [Å]
3 4 5 6 7
g
Resolution distribution at FSC cutoff 0.5
Elevation
Azimuth
Resolution [Å]

h

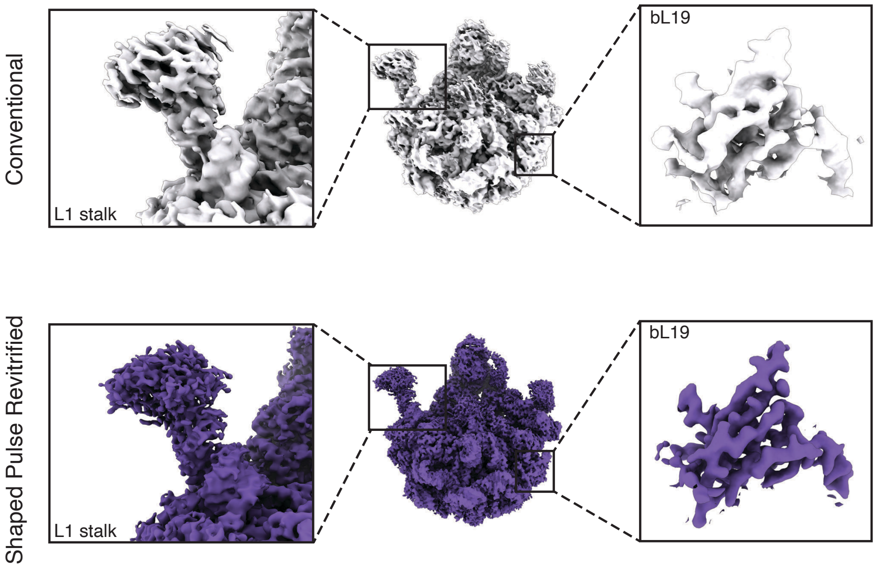

Supplementary Figure 8 | Cryo-EM data processing workflow for a shaped pulse revitrified sample of the 50S ribosome. a Representative micrograph. Scale bar 500 Å. **b** 2D class averages **c** Data processing workflow in cryoSPARC. The symmetry applied is indicated in parentheses. A resolution of 2.9 Å was obtained in the final reconstruction. The unsharpened map is shown at 3.5 σ. **d** Three-dimensional representation of angular distribution of the particles, with the color and height of the cylinders representing the frequency of observation (red: more abundant, blue: less abundant). **e** Plot of the 3DFSC with the 0.143 cutoff indicated (grey line). The mean FSC value is shown as a solid blue line, with dark blue shading corresponding to one standard deviation, and the light blue shading indicating the minimum and maximum values. The green bars represent a histogram of the resolution values obtained from the directional FSC. The narrow distribution of the minimum and maximum 0.143 crossing reflect the improvement of the preferred orientation of the 50S ribosomal subunit. **f** Final map with the local resolution estimation indicated in color. **g** The resolution distribution plot shown at FSC cutoff of 0.5, as obtained from the 3DFSC job in cryoSPARC. Compared to the control, the directional resolution distribution improved significantly (Fig. S8g). **h** Comparison of the reconstructions from the conventional sample with the shaped pulse revitrified sample. The bL19 subunit is contoured at 6 σ.

3 | Cryo-EM data processing – HIV-1 Envelope ectodomain protein

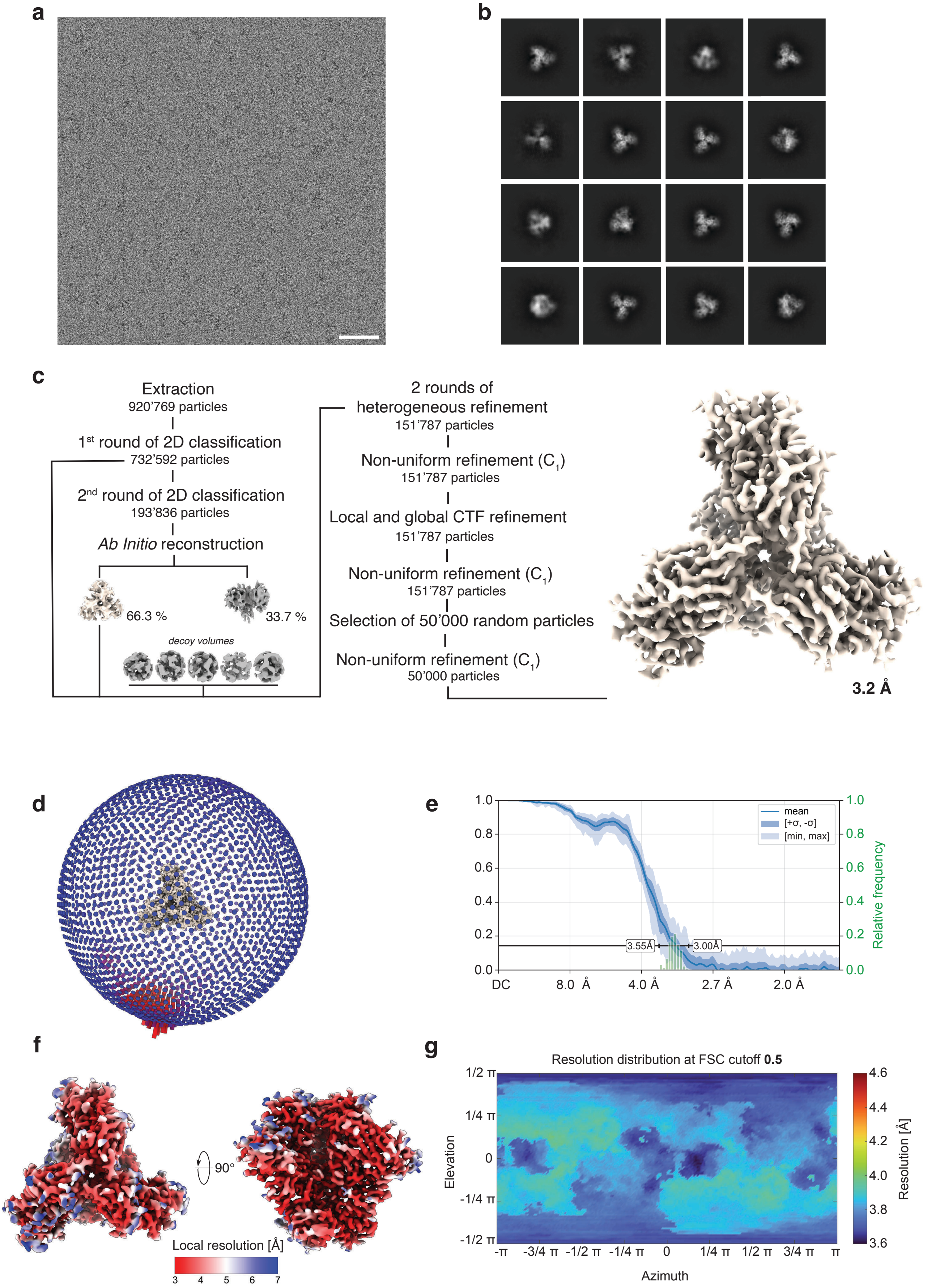

Supplementary Figure 9 | Cryo-EM data processing workflow for a conventional sample of the HIV-1 Envelope ectodomain protein. a Representative micrograph. Scale bar 100 Å. **b** 2D class averages. **c** Data processing workflow in cryoSPARC. The symmetry applied is indicated in parentheses. A resolution of 3.2 Å was obtained in the final reconstruction. The map is shown at 15 σ. **d** Three-dimensional representation of angular distribution of the particles, with the color and height of the cylinders representing the frequency of observation (red: more abundant, blue: less abundant). **e** Plot of the 3DFSC with the 0.143 cutoff indicated (grey line). The mean FSC value is shown as a solid blue line, with dark blue shading corresponding to one standard deviation, and the light blue shading indicating the minimum and maximum values. The green bars represent a histogram of the resolution values obtained from the directional FSC. **f** Final map with the local resolution estimation indicated in color. **g** The resolution distribution plot shown at FSC cutoff of 0.5, as obtained from the 3DFSC job in cryoSPARC.

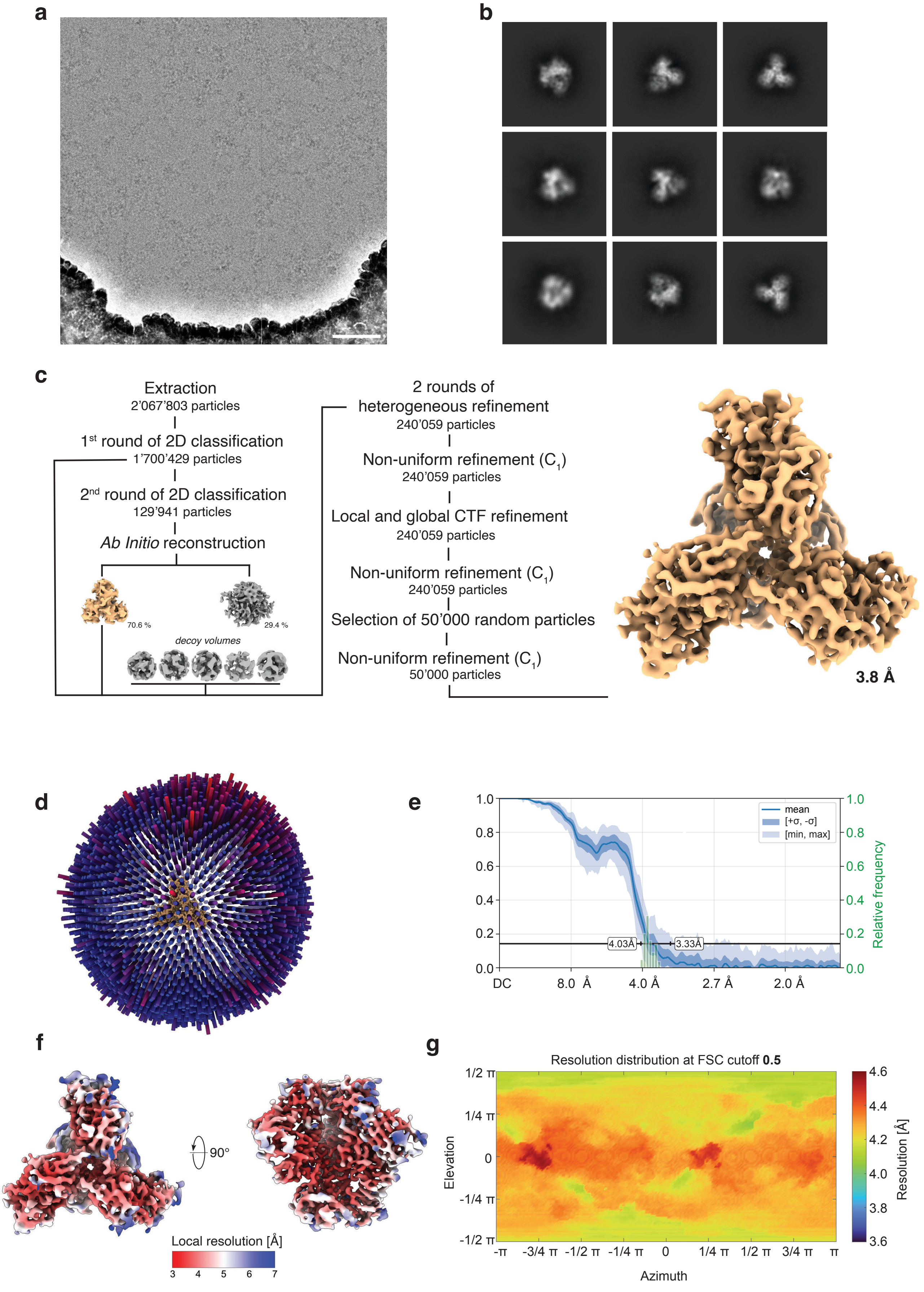
a
b
c
Extraction
2'067'803 particles
1st round of 2D classification
1'700'429 particles
2nd round of 2D classification
129'941 particles
Ab Initio reconstruction
70.6 %
29.4 %
decoy volumes
2 rounds of
heterogeneous refinement
240'059 particles
Non-uniform refinement (C1)
240'059 particles
Local and global CTF refinement
240'059 particles
Non-uniform refinement (C1)
240'059 particles
Selection of 50'000 random particles
Non-uniform refinement (C1)
50'000 particles
3.8 Å
d
e
mean
[+σ, -σ]
[min, max]
4.03Å
3.33Å
Relative frequency
DC
8.0 Å
4.0 Å
2.7 Å
2.0 Å
f
90°
Local resolution [Å]
3
4
5
6
7
g
Resolution distribution at FSC cutoff 0.5
Elevation
Azimuth
Resolution [Å]
1/2 π
1/4 π
0
-1/4 π
-1/2 π
-π
-3/4 π
-1/2 π
-1/4 π
1/4 π
1/2 π
3/4 π
π
4.6
4.4
4.2
4.0
3.8
3.6

h

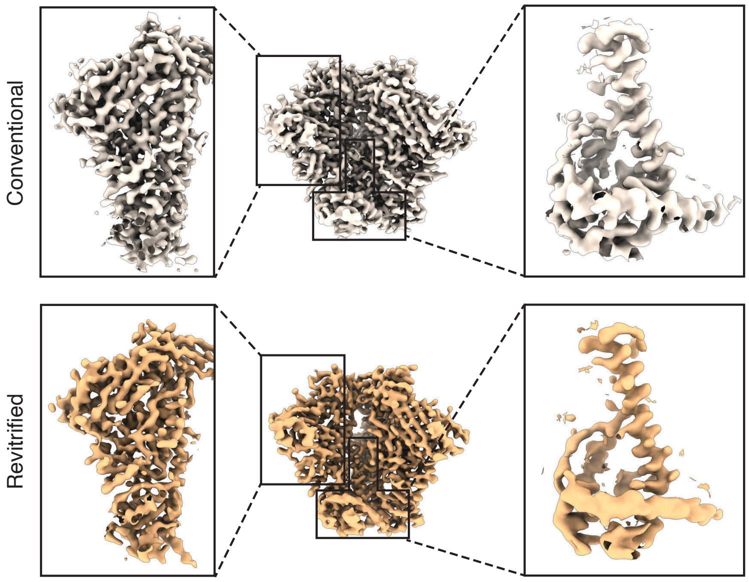

Supplementary Figure 10 | Cryo-EM data processing workflow for a revitrified sample of the HIV-1 Envelope ectodomain protein. a Representative micrograph. Scale bar 100 Å. **b** 2D class averages. **c** Data processing workflow in cryoSPARC. The symmetry applied is indicated in parentheses. A resolution of 3.8 Å was obtained in the final reconstruction. The map is contoured at 15 σ. **d** Three-dimensional representation of angular distribution of the particles, with the color and height of the cylinders representing the frequency of observation (red: more abundant, blue: less abundant). **e** Plot of the 3DFSC with the 0.143 cutoff indicated (grey line). The mean FSC value is shown as a solid blue line, with dark blue shading corresponding to one standard deviation, and the light blue shading indicating the minimum and maximum values. The green bars represent a histogram of the resolution values obtained from the directional FSC. **f** Final map with the local resolution estimation indicated in color. **g** The resolution distribution plot shown at FSC cutoff of 0.5, as obtained from the 3DFSC job in cryoSPARC. Despite the improvement in angular distribution, the resolution decreased in the revitrified sample, which we attributed to the presence of astigmatism in the microscope during data collection. **h** Comparison of the reconstructions from the conventional sample with the revitrified sample.

4 | Cryo-EM data processing – Hemagglutinin

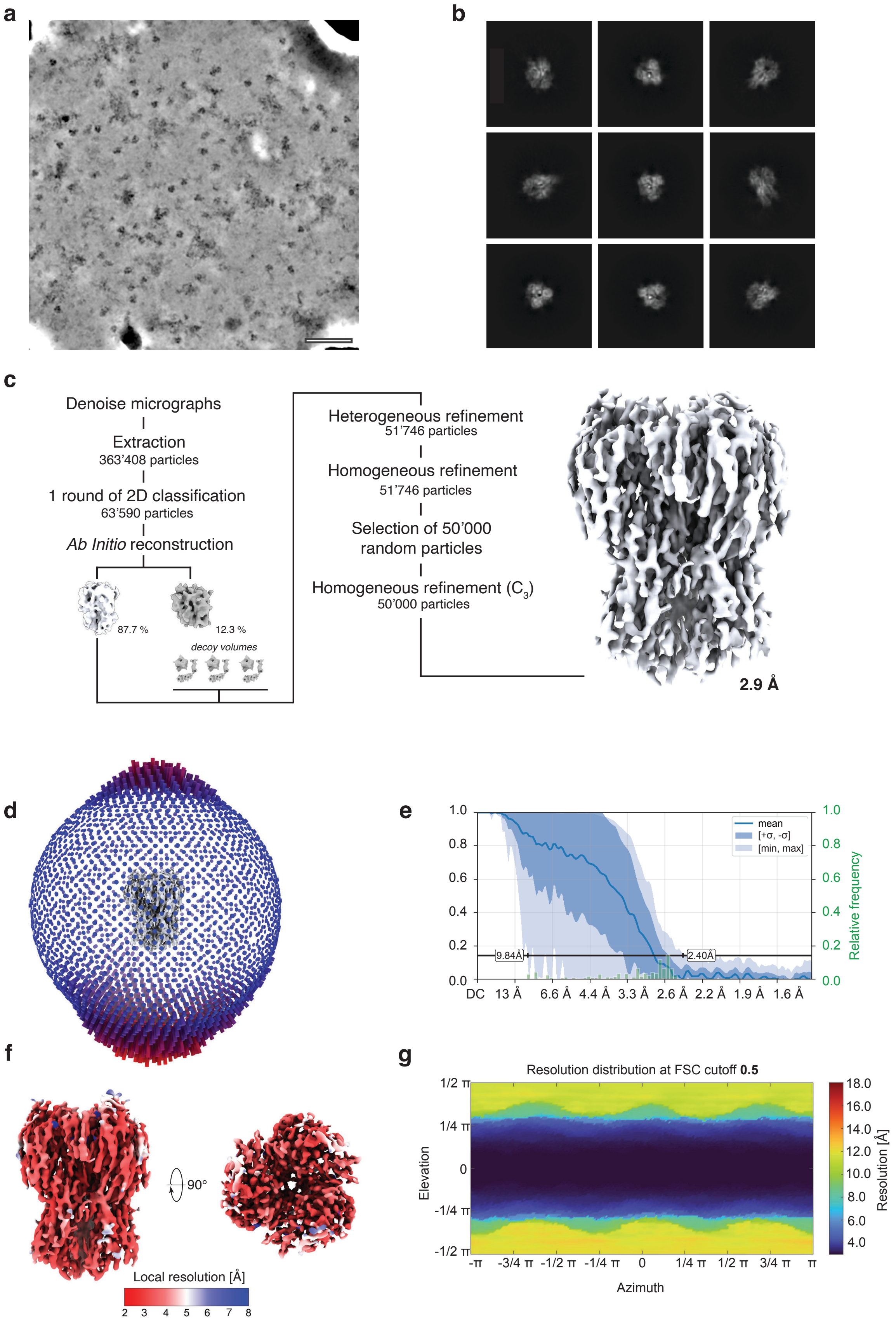

Supplementary Figure 11 | Cryo-EM data processing workflow for a conventional sample of Hemagglutinin. a Representative denoised micrograph. Scale bar 100 Å. **b** 2D class averages. **c** Data processing workflow in cryoSPARC. The final map reconstructed to 2.9 Å (as reported by cryoSPARC. The apparent resolution, due to anisotropy is worse). The symmetry applied is indicated in parentheses. The map is shown 7 σ. **d** Three-dimensional representation of angular distribution of the particles, with the color and height of the cylinders representing the frequency of observation (red: more abundant, blue: less abundant). **e** Plot of the 3DFSC with the 0.143 cutoff indicated (grey line). The mean FSC value is shown as a solid blue line, with dark blue shading corresponding to one standard deviation, and the light blue shading indicating the minimum and maximum values. The green bars represent a histogram of the resolution values obtained from the directional FSC. **f** Final map with the local resolution estimation indicated in color. **g** The resolution distribution plot shown at FSC cutoff of 0.5, as obtained from the 3DFSC job in cryoSPARC.

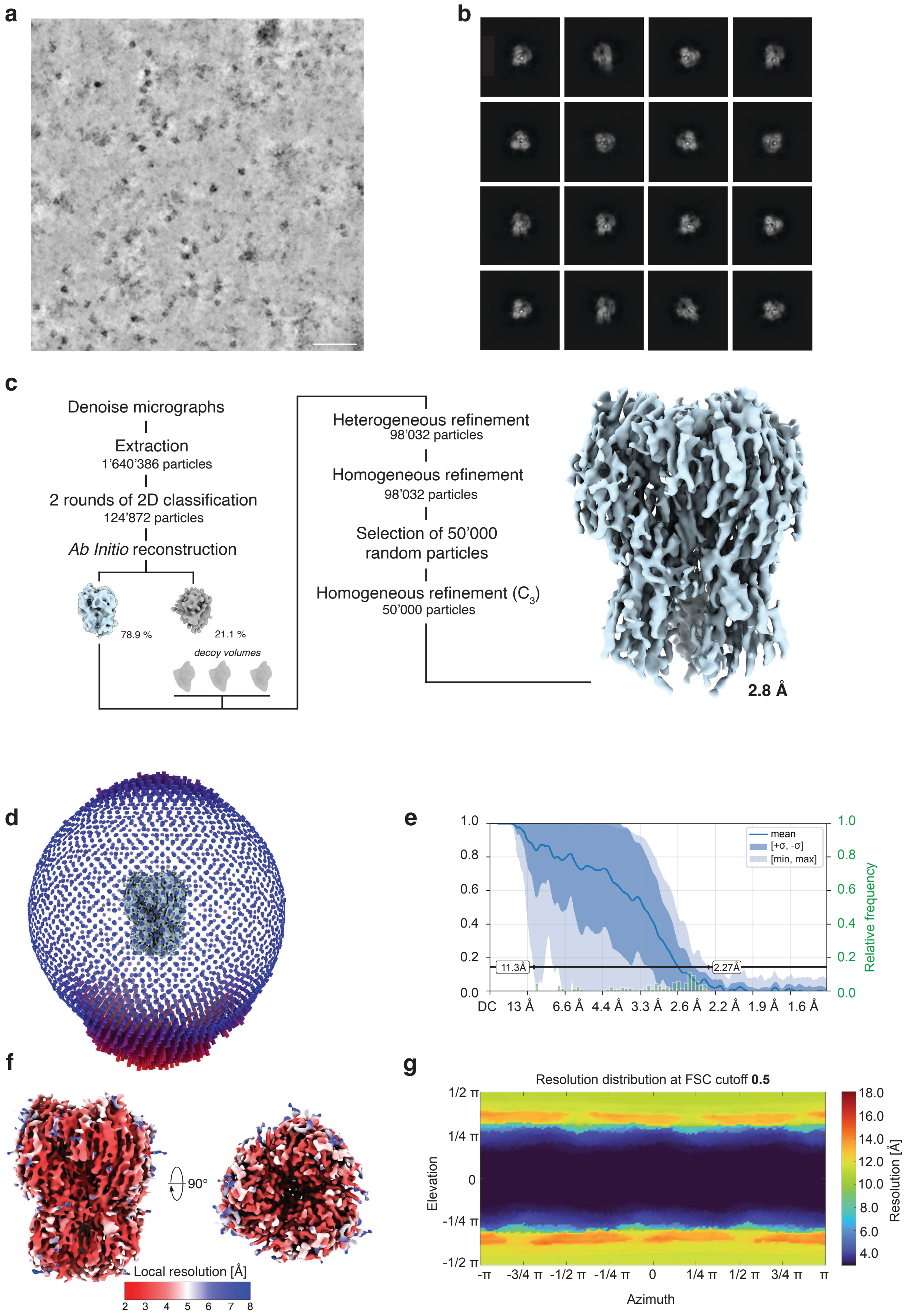
a
b
c
Denoise micrographs
Extraction
1'640'386 particles
2 rounds of 2D classification
124'872 particles
Ab Initio reconstruction
78.9 %
21.1 %
decoy volumes
Heterogeneous refinement
98'032 particles
Homogeneous refinement
98'032 particles
Selection of 50'000
random particles
Homogeneous refinement (C3)
50'000 particles
2.8 Å
d
e
mean
[+σ, -σ]
[min, max]
11.3Å
2.27Å
Relative frequency
DC 13 Å 6.6 Å 4.4 Å 3.3 Å 2.6 Å 2.2 Å 1.9 Å 1.6 Å
f
90°
Local resolution [Å]
2 3 4 5 6 7 8
g
Resolution distribution at FSC cutoff 0.5
Elevation
Azimuth
Resolution [Å]

Supplementary Figure 12 | Cryo-EM data processing workflow for a revitrified sample of Hemagglutinin. a Representative denoised micrograph. Scale bar 100 Å. **b** 2D class averages **c** Data processing workflow in cryoSPARC. The final map reconstructed to 2.8 Å (as reported by cryoSPARC. The apparent resolution, due to anisotropy is worse). The symmetry applied is indicated in parentheses. The map is shown at 7 σ. **d** Three-dimensional representation of angular distribution of the particles, with the color and height of the cylinders representing the frequency of observation (red: more abundant, blue: less abundant). **e** Plot of the 3DFSC with the 0.143 cutoff indicated (grey line). The mean FSC value is shown as a solid blue line, with dark blue shading corresponding to one standard deviation, and the light blue shading indicating the minimum and maximum values. The green bars represent a histogram of the resolution values obtained from the directional FSC. **f** Final map with the local resolution estimation indicated in color. **g** The resolution distribution plot shown at FSC cutoff of 0.5, as obtained from the 3DFSC job in cryoSPARC.

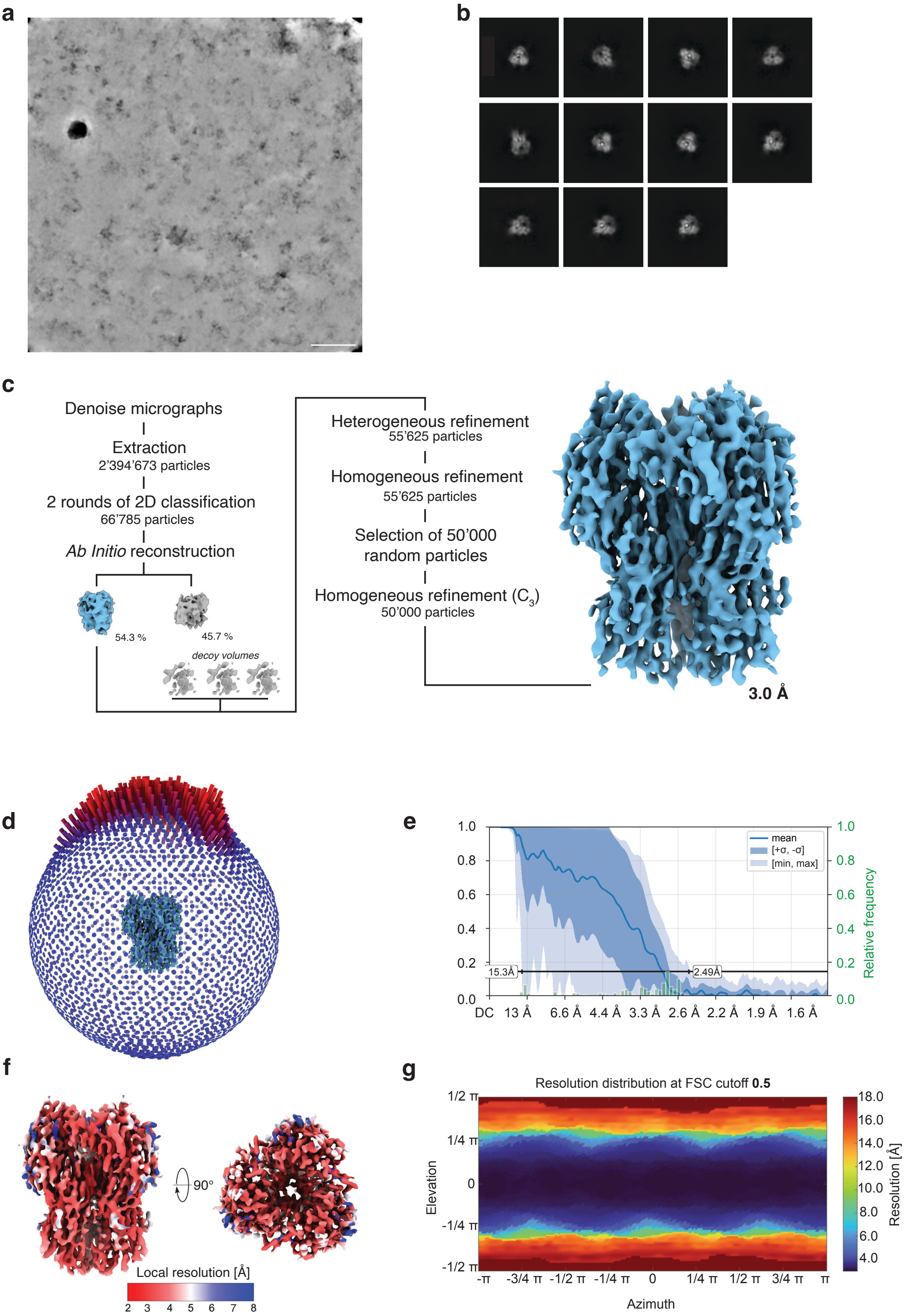
a
b
c
Denoise micrographs
Extraction
2'394'673 particles
2 rounds of 2D classification
66'785 particles
Ab Initio reconstruction
54.3 %
45.7 %
decoy volumes
Heterogeneous refinement
55'625 particles
Homogeneous refinement
55'625 particles
Selection of 50'000
random particles
Homogeneous refinement (C_3)
50'000 particles
3.0 Å
d
e
mean
[+σ, -σ]
[min, max]
Relative frequency
15.3Å
2.49Å
DC 13 Å 6.6 Å 4.4 Å 3.3 Å 2.6 Å 2.2 Å 1.9 Å 1.6 Å
f
90°
Local resolution [Å]
2 3 4 5 6 7 8
g
Resolution distribution at FSC cutoff 0.5
Elevation
Azimuth
Resolution [Å]

Supplementary Figure 13 | Cryo-EM data processing workflow for a deposited and revitrified sample of Hemagglutinin. **a** Representative denoised micrograph. Scale bar 100 Å. **b** 2D class averages. **c** Data processing workflow in cryoSPARC. The final map reconstructed to 3.0 Å (as reported by cryoSPARC. The apparent resolution, due to anisotropy is worse). The symmetry applied is indicated in parentheses. The map is shown at 7 σ. **d** Three-dimensional representation of angular distribution of the particles, with the color and height of the cylinders representing the frequency of observation (red: more abundant, blue: less abundant). **e** Plot of the 3DFSC with the 0.143 cutoff indicated (grey line). The mean FSC value is shown as a solid blue line, with dark blue shading corresponding to one standard deviation, and the light blue shading indicating the minimum and maximum values. The green bars represent a histogram of the resolution values obtained from the directional FSC. **f** Final map with the local resolution estimation indicated in color. **g** The resolution distribution plot shown at FSC cutoff of 0.5, as obtained from the 3DFSC job in cryoSPARC. The increase in ice thickness after deposition leads to a decrease in the resolution distribution.

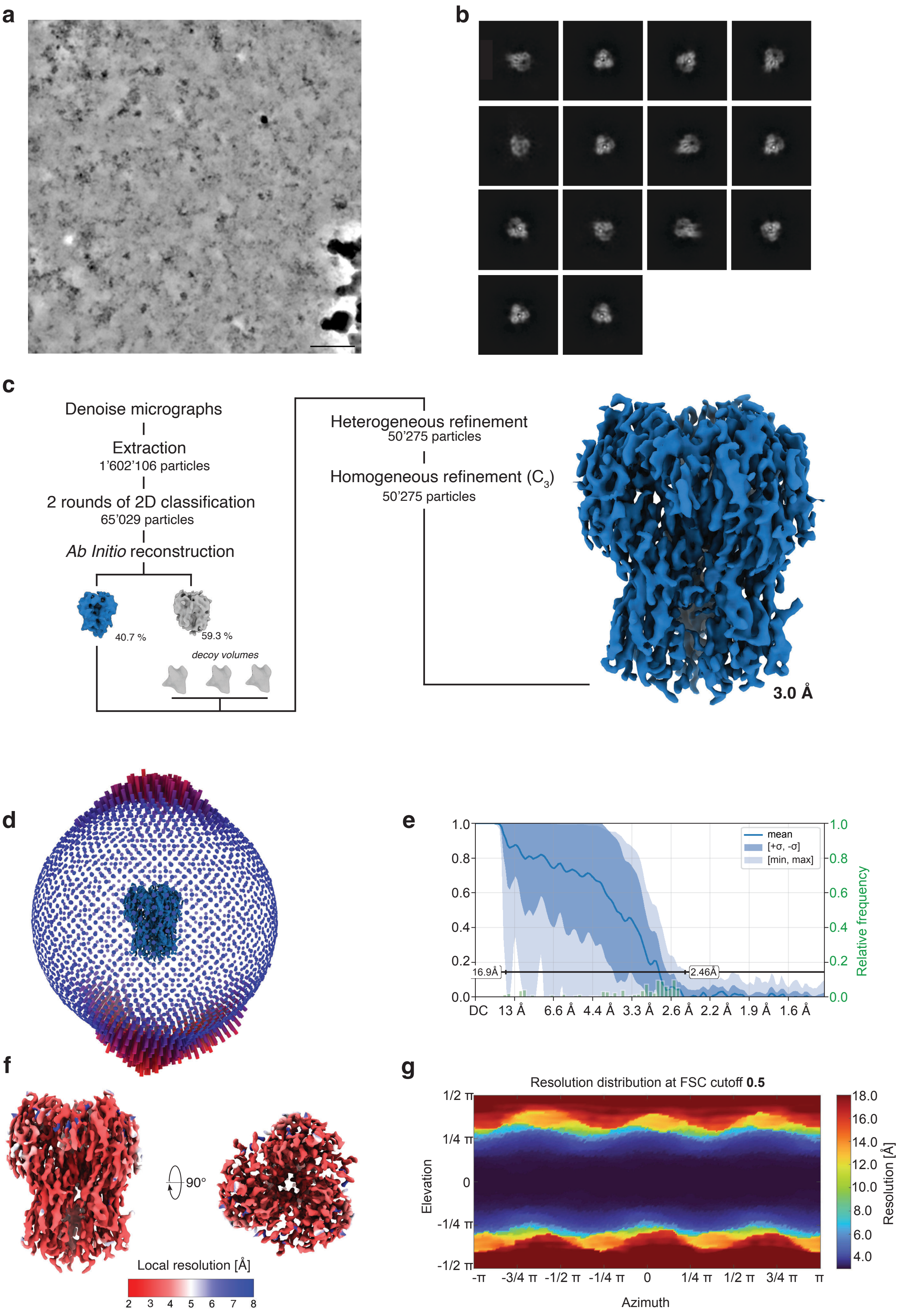
a
b
c
Denoise micrographs
Extraction
1'602'106 particles
2 rounds of 2D classification
65'029 particles
Ab Initio reconstruction
40.7 %
59.3 %
decoy volumes
Heterogeneous refinement
50'275 particles
Homogeneous refinement (C3)
50'275 particles
3.0 Å
d
e
mean
[+σ, -σ]
[min, max]
Relative frequency
16.9Å
2.46Å
DC 13 Å 6.6 Å 4.4 Å 3.3 Å 2.6 Å 2.2 Å 1.9 Å 1.6 Å
f
90°
Local resolution [Å]
2 3 4 5 6 7 8
g
Resolution distribution at FSC cutoff 0.5
Elevation
Azimuth
Resolution [Å]

Supplementary Figure 14 | Cryo-EM data processing workflow for a shaped pulse revitrified sample of Hemagglutinin. **a** Representative denoised micrograph. Scale bar 100 Å. **b** 2D class averages. **c** Data processing workflow in cryoSPARC. The final map reconstructed to 3.0 Å (as reported by cryoSPARC. The apparent resolution, due to anisotropy is worse). The symmetry applied is indicated in parentheses. The map is shown at 7 σ. **d** Three-dimensional representation of angular distribution of the particles, with the color and height of the cylinders representing the frequency of observation (red: more abundant, blue: less abundant). **e** Plot of the 3DFSC with the 0.143 cutoff indicated (grey line). The mean FSC value is shown as a solid blue line, with dark blue shading corresponding to one standard deviation, and the light blue shading indicating the minimum and maximum values. The green bars represent a histogram of the resolution values obtained from the directional FSC. **f** Final map with the local resolution estimation indicated in color. **g** The resolution distribution plot shown at FSC cutoff of 0.5, as obtained from the 3DFSC job in cryoSPARC.

5 | Simulation of the temperature evolution of the sample

The temperature evolution of the sample under laser irradiation was simulated with COMSOL Multiphysics 6.1, as previously described [1,2]. Figure S15a schematically displays the shape of a rectangular pulse as used in the experiments of Figs. 1 and 3 together with the resulting temperature evolution of the sample. We report the average sample temperature within a hole in the gold film in the center of the laser spot. Here, a laser power of 150 mW was simulated, for which the sample temperature reaches 285 K at the end of the laser pulse. For comparison, Fig. S15b shows the simulated temperature evolution of the sample for a shaped pulse with an intense leading edge as used in the experiments of Fig. 2 (1 μs duration of the initial spike with 10 times the laser power). The simulation revealed that the sample temperature rises more rapidly at the beginning of the laser pulse and overshoots the plateau temperature for a duration of approximately 1.5 μs, reaching a maximum of approximately 310 K. Note that while schematic laser pulse shapes are shown in Fig. S16, experimentally determined pulse shapes were used in the simulations as recorded with a fast photodiode.

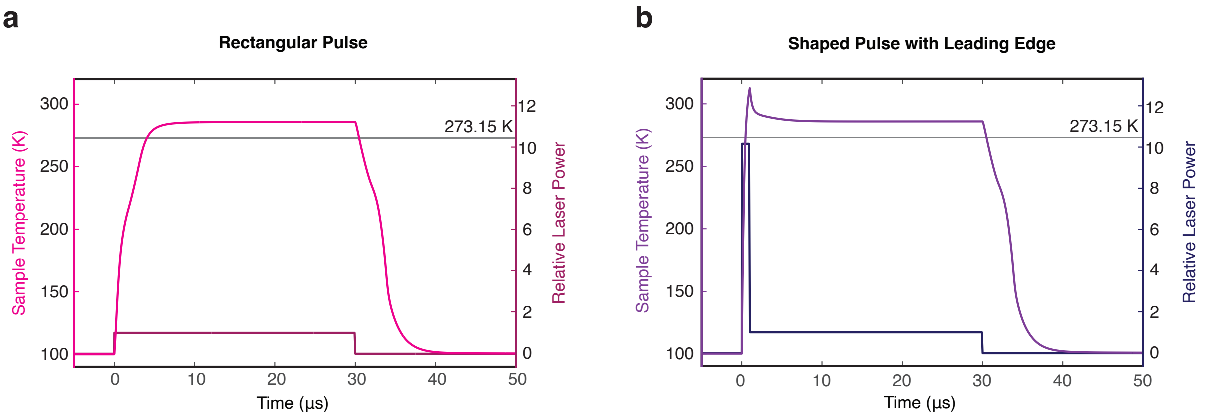

Supplementary Figure 15 | Simulation of the sample temperature evolution under irradiation with a rectangular and a shaped laser pulse. a Simulated temperature evolution of the sample (pink) under irradiation with a rectangular laser pulse (schematically shown in magenta). **b** Under irradiation with a shaped laser pulse (navy, 1 μs initial spike of 10 times the power), the sample heats up more rapidly, and the temperature briefly overshoots (shown in purple).

6 | Cryo-EM data collection, refinement and validation statistics

Supplementary Table 1 | Cryo-EM statistics for the datasets collected on the T20S proteasome

	T20S conventional<br><br>EMDB-51744<br>EMPIAR-12389	T20S revitrified<br><br>EMDB-51745<br>EMPIAR-12388	T20S revitrified after deposition<br><br>EMDB-51746<br>EMPIAR-12390
**Data collection and processing**			
Microscope	Titan Krios G3i	Titan Krios G3i	Titan Krios G3i
Camera	K3	K3	K3
Energy Filter (slit)	BioQuantum (20 eV)	BioQuantum (20 eV)	BioQuantum (20 eV)
Magnification	130'000	130'000	130'000
Voltage (kV)	300	300	300
Electron exposure (e^{-}/Å^2)	67.7	65.3	67.7
Defocus range (µm)	-0.6 to -2.0	-0.6 to -2.0	-0.6 to -2.0
Pixel size (Å)	0.651	0.651	0.651
Symmetry imposed	D_7	D_7	D_7
Initial particle images (no.)	397’944	450’862	409’644
Final particle images (no.)	50’000	50’000	50’000
Map resolution (Å)	2.6	2.5	2.8
FSC threshold	0.143	0.143	0.143
Map sharpening *B* factor (Å^2)	82.7	79.7	95.0
Orientation Diagnostics			
cFAR	0.82	0.85	0.83
SCF*	0.88	0.95	0.97

Supplementary Table 2 | Cryo-EM statistics for the datasets collected on the 50S ribosomal subunit

	50S conventional<br><br>EMDB-51747<br>EMPIAR-12397	50S revitrified<br><br>EMDB-51748<br>EMPIAR-12398	50S revitrified after deposition<br><br>EMDB-51749<br>EMPIAR-12399	50S shaped pulse conventional<br><br>EMDB-51750<br>EMPIAR-12435	50S shaped pulse revitrified<br><br>EMDB-51751<br>EMPIAR-12436
**Data collection and processing**					
Microscope	Titan Krios G4	Titan Krios G4	Titan Krios G4	Titan Krios G3	Titan Krios G3
Camera	Falcon 4i	Falcon 4i	Falcon 4i	K3	K3
Energy Filter (slit)	none	none	none	BioQuantum (20 eV)	BioQuantum (20 eV)
Magnification	120'000	120'000	120'000	130'000	130'000
Voltage (keV)	300	300	300	300	300
Electron exposure ($e^-/Å^2$)	38.6	40.0	40.0	61.8	63.4
Defocus range (μm)	-0.4 to -1.0	-0.4 to -1.0	-0.4 to -1.0	-0.4 to -1.6	-0.4 to -1.6
Pixel size (Å)	0.658	0.658	0.658	0.651	0.651
Symmetry imposed	C_1	C_1	C_1	C_1	C_1
Initial particle images (no.)	296’144	242’533	275’189	373’837	342’718
Final particle images (no.)	50’000	50’000	50’000	50’000	50’000
Map resolution (Å)	3.5	3.2	3.4	4.1	2.9
FSC threshold	0.143	0.143	0.143	0.143	0.143
Map sharpening *B* factor ($Å^2$)	43.6	47.9	37.7	57.8	40.4
Orientation Diagnostics					
cFAR	0.10	0.51	0.50	0.03	0.70
SCF*	0.42	0.74	0.90	0.18	0.90

Supplementary Table 3 | Cryo-EM statistics for the datasets collected on the HIV-1 Envelope ectodomain protein

	HIV-1 conventional<br><br>EMDB-51752<br>EMPIAR-12437	HIV-1 revitrified<br><br>EMDB-51753<br>EMPIAR-12438
**Data collection and processing**		
Microscope	Titan Krios G4	Titan Krios G4
Camera	Falcon 4i	Falcon 4i
Energy Filter	none	none
Magnification	96'000	96'000
Voltage (kV)	300	300
Electron exposure (e^{-}/Å^2)	50.0	50.0
Defocus range (μm)	-1.0 to -2.4	-1.0 to -2.4
Pixel size (Å)	0.830	0.830
Symmetry imposed	C_1	C_1
Initial particle images (no.)	920’769	2’067’803
Final particle images (no.)	50’000	50’000
Map resolution (Å)	3.2	3.8
FSC threshold	0.143	0.143
Map sharpening *B* factor (Å^2)	21.1	29.7
Orientation Diagnostics		
cFAR	0.78	0.71
SCF*	0.89	0.99

Supplementary Table 4 | Cryo-EM Statistics for the datasets collected on Hemagglutinin (HA)

	HA conventional<br><br>EMDB-51754<br>EMPIAR-12439	HA revitrified<br><br>EMDB-51755<br>EMPIAR-12440	HA revitrified after deposition<br><br>EMDB-51756<br>EMPIAR-12441	HA shaped pulse revitrified<br><br>EMDB-51757<br>EMPIAR-12442
**Data collection and processing**				
Microscope	Titan Krios G4	Titan Krios G4	Titan Krios G4	Titan Krios G4
Camera	Falcon 4i	Falcon 4i	Falcon 4i	Falcon 4i
Energy Filter	Selectris X (10 eV)	Selectris X (10 eV)	Selectris X (10 eV)	Selectris X (10 eV)
Magnification	165'000	165'000	165'000	165'000
Voltage (kV)	300	300	300	300
Electron exposure ($e^{-}/Å^2$)	40.0	40.0	40.0	40.0
Defocus range (μm)	-0.8 to -2.5	-0.8 to -2.5	-0.8 to -2.5	-0.8 to -2.5
Pixel size (Å)	0.732	0.732	0.732	0.732
Symmetry imposed	C_3	C_3	C_3	C_3
Initial particle images (no.)	363’408	1’352’936	2’394’673	1’602’206
Final particle images (no.)	50’000	50’000	50’000	50’000
Map resolution (Å)	(2.9)	(2.8)	(3.0)	(3.0)
FSC threshold	0.143	0.143	0.143	0.143
Map sharpening *B* factor ($Å^2$)	73.1	60.2	68.1	75.6
Orientation Diagnostics				
cFAR	0.02	0.02	0.03	0.01
SCF*	0.60	0.56	0.58	0.55

7 | References

1. Krüger, C. R., Mowry, N. J., Bongiovanni, G., Drabbels, M. & Lorenz, U. J. Electron diffraction of deeply supercooled water in no man's land. *Nat. Commun.* **14**, 2812 (2023).
2. Mowry, N. J., Krüger, C. R., Bongiovanni, G., Drabbels, M. & Lorenz, U. J. Flash melting amorphous ice. *J. Chem. Phys.* **160**, 184502 (2024).
