# Supplementary material for: Laser flash melting cryo-EM samples to overcome preferred orientation
Source: Nat Methods. 2025 Aug 28;22(9):1880–6. doi: 10.1038/s41592-025-02796-y (PMC12446059; doi:10.1038/s41592-025-02796-y)
Supplement: Supplementary file 2 — Reporting Summary [file 41592_2025_2796_MOESM2_ESM.pdf]

nature portfolio

Corresponding author(s): Ulrich J. Lorenz

Last updated by author(s): 02/06/2025

Reporting Summary

Nature Portfolio wishes to improve the reproducibility of the work that we publish. This form provides structure for consistency and transparency in reporting. For further information on Nature Portfolio policies, see our Editorial Policies and the Editorial Policy Checklist.

Statistics

For all statistical analyses, confirm that the following items are present in the figure legend, table legend, main text, or Methods section.

n/a	Confirmed	
☐	☒	The exact sample size (*n*) for each experimental group/condition, given as a discrete number and unit of measurement
☒	☐	A statement on whether measurements were taken from distinct samples or whether the same sample was measured repeatedly
☒	☐	The statistical test(s) used AND whether they are one- or two-sided<br>*Only common tests should be described solely by name; describe more complex techniques in the Methods section.*
☒	☐	A description of all covariates tested
☒	☐	A description of any assumptions or corrections, such as tests of normality and adjustment for multiple comparisons
☐	☒	A full description of the statistical parameters including central tendency (e.g. means) or other basic estimates (e.g. regression coefficient) AND variation (e.g. standard deviation) or associated estimates of uncertainty (e.g. confidence intervals)
☒	☐	For null hypothesis testing, the test statistic (e.g. *F*, *t*, *r*) with confidence intervals, effect sizes, degrees of freedom and *P* value noted<br>*Give P values as exact values whenever suitable.*
☒	☐	For Bayesian analysis, information on the choice of priors and Markov chain Monte Carlo settings
☒	☐	For hierarchical and complex designs, identification of the appropriate level for tests and full reporting of outcomes
☒	☐	Estimates of effect sizes (e.g. Cohen's *d*, Pearson's *r*), indicating how they were calculated

Our web collection on statistics for biologists contains articles on many of the points above.

Software and code

Policy information about availability of computer code

Data collection	*Provide a description of all commercial, open source and custom code used to collect the data in this study, specifying the version used OR state that no software was used.*
Data analysis	cryoSPARC v.4.4 - v.4.6, ChimeraX v1.7-1.8

For manuscripts utilizing custom algorithms or software that are central to the research but not yet described in published literature, software must be made available to editors and reviewers. We strongly encourage code deposition in a community repository (e.g. GitHub). See the Nature Portfolio guidelines for submitting code & software for further information.

Data

Policy information about availability of data

All manuscripts must include a data availability statement. This statement should provide the following information, where applicable:

- Accession codes, unique identifiers, or web links for publicly available datasets
- A description of any restrictions on data availability
- For clinical datasets or third party data, please ensure that the statement adheres to our policy

The data that support the findings of this study are available from the corresponding author upon request. The cryo-EM maps have been deposited in the Electron Microscopy Data Bank (EMDB) and the Electron Microscopy Public Image Archive (EMPIAR) under accession codes EMD-51744 and EMPIAR-12389 (T20S conventional), EMD-51745 and EMPIAR-12388 (T20S revitrified), EMD-51746 and EMPIAR-12390 (T20S revitrified after deposition), EMD-51747 and EMPIAR-12397

(50S conventional), EMD-51748 and EMPIAR-12398 (50S revitrified), EMD-51749 and EMPIAR-12399 (50S revitrified after deposition), EMD-51750 and EMPIAR-12435 (50S shaped pulse conventional), EMD-51751 and EMPIAR-12436 (50S shaped pulse revitrified), EMD-51752 and EMPIAR-12437 (HIV conventional), EMD-51753 and EMPIAR-12438 (HIV revitrified), EMD-51754 and EMPIAR-12439 (HA conventional), EMD-51755 and EMPIAR-12440 (HA revitrified), EMD-51756 and EMPIAR-12441 (HA revitrified after deposition), and EMD-51757 and EMPIAR-12442 (HA shaped pulse revitrified).

Human research participants

Policy information about studies involving human research participants and Sex and Gender in Research.

Reporting on sex and gender	No such information was collected.
Population characteristics	see above
Recruitment	No participants were recruited for this study.
Ethics oversight	No ethics organization needed to oversee the study.

Note that full information on the approval of the study protocol must also be provided in the manuscript.

Field-specific reporting

Please select the one below that is the best fit for your research. If you are not sure, read the appropriate sections before making your selection.

☒ Life sciences ☐ Behavioural & social sciences ☐ Ecological, evolutionary & environmental sciences

For a reference copy of the document with all sections, see nature.com/documents/nr-reporting-summary-flat.pdf

Life sciences study design

All studies must disclose on these points even when the disclosure is negative.

Sample size	No sample size calculations were performed. 50'000 random particles were chosen for each reconstruction to have an equal number of particles per reconstruction. This allowed for comparisons of the orientation distribution between the experimental conditions.
Data exclusions	Particles , which did not contain any useful information, were excluded during 2D classification and 3D classification through ab initio reconstruction and heterogeneous refinement. The exclusion was performed by assessment of the classification results (whether the classes correspond to the protein of interest or not).
Replication	The applicability of the methods was tested on two to four different and independent protein systems. Each protein system was investigated once.
Randomization	No randomization was performed, as there were no experimental groups in this study.
Blinding	No blinding was performed, as there were no experimental groups in this study.

Reporting for specific materials, systems and methods

We require information from authors about some types of materials, experimental systems and methods used in many studies. Here, indicate whether each material, system or method listed is relevant to your study. If you are not sure if a list item applies to your research, read the appropriate section before selecting a response.

Materials & experimental systems

n/a	Involved in the study	
☒	☐	Antibodies
☒	☐	Eukaryotic cell lines
☒	☐	Palaeontology and archaeology
☒	☐	Animals and other organisms
☒	☐	Clinical data
☒	☐	Dual use research of concern

Methods

n/a	Involved in the study	
☒	☐	ChIP-seq
☒	☐	Flow cytometry
☒	☐	MRI-based neuroimaging
